# How Does the Rate of Chain Exchange Relate to Stress Relaxation in Triblock Copolymer Networks?

**DOI:** 10.1021/acscentsci.4c02031

**Published:** 2025-02-20

**Authors:** Joanna M. White, Taehyoung Kim, Frank S. Bates, Timothy P. Lodge

**Affiliations:** † Department of Chemical Engineering and Materials Science, University of Minnesota, Minneapolis, Minnesota 55455, United States; ‡ Department of Chemistry, University of Minnesota, Minneapolis, Minnesota 55455, United States

## Abstract

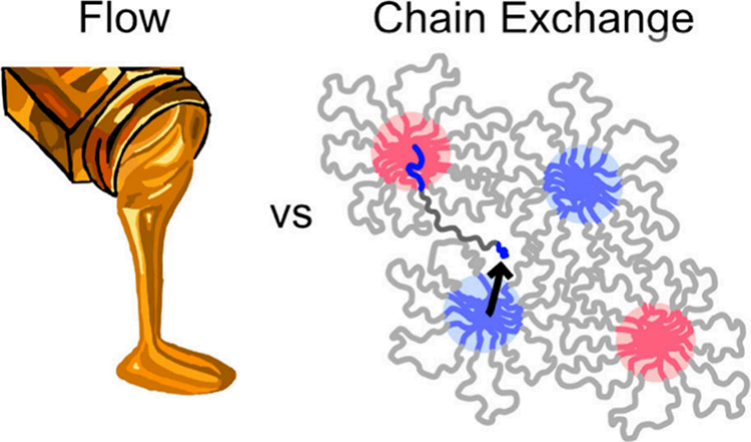

The relationship between macroscopic stress relaxation and molecular-level chain exchange in triblock copolymer micelles has been explored using rheology and time-resolved small-angle neutron scattering (TR-SANS), marking the first measurements of chain exchange in concentrated triblock networks. It has long been assumed in models of transient or thermoreversible networks that the time scales for these two processes are equal. Experimentally, we find that stress relaxation occurs many orders-of-magnitude faster than chain exchange. This difference is quantitatively explained by modest dispersity in the core block that results in a slight asymmetry within any given nominally symmetric triblock. For stress relaxation to occur, only the shorter chain must pull out, while chain exchange is slowed due to the requirement of the eventual pullout of the longer block. The pullout time is extremely sensitive to the length of the core block. This mechanism is supported by measurements with an intentionally asymmetric triblock copolymer, which displays an even larger difference between the stress relaxation and chain exchange rates. These results establish a quantitative molecular-level picture of the chain dynamics associated with stress relaxation in triblock copolymer networks.

## Abstract 2

A quantitative relationship is established between the flow time of a BAB triblock copolymer network, from rheology, and the time for a single B end block to escape from its micellar cross-link, determined by neutron scattering.

## Introduction

Block copolymers represent a broadly tunable, facile route to nanostructured materials.^1−4^ While AB diblocks and BAB triblocks readily assemble into similar morphologies, the triblock architecture offers huge advantages due to enhanced mechanical properties. These arise from a network structure; the B end-blocks assemble into spherical micellar cross-links, while bridging A blocks confer substantial elasticity.^5−16^ Furthermore, at temperatures above the B block glass transition, such materials are reprocessable, captured by their designation as “thermoplastic elastomers” (TPE).^17−20^ Over the past 50 years the TPE market has grown to several billion dollars, with applications ranging from athletic footwear and hot-melt adhesives to hydrogels for controlled biologic release and coatings for drug-eluting stents.^17−20^

**Figure 1:**
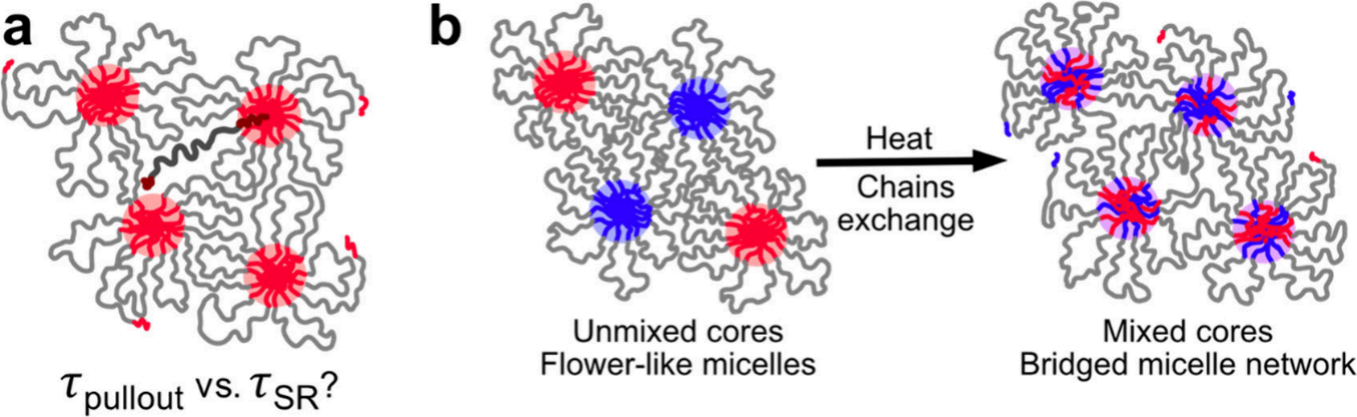
BAB triblock copolymers can self-assemble into networks of bridged micelles. (a) A persistent question is how the molecular-level rate of chain pullout (τ_pullout_) relates to the macroscopic rate of stress relaxation (τ_SR_) measured using rheology. (b) Design of time-resolved small-angle neutron scattering (TR-SANS) measurements for concentrated triblock solutions. Flower-like micelles with distinct deuterium-labeled (red) and unlabeled (blue) cores are mixed. Upon annealing at elevated temperatures, chains exchange and the cores mix.

In any thermoreversible polymer network, there is a longest viscoelastic relaxation time, or stress relaxation time, τ_SR_, that determines the time scale of reprocessability as a function of temperature. There is also an average lifetime for a cross-link, which in the TPE case is assumed to be correlated with the time scale for escape of a B block from a given micellar core, τ_pullout_.^11−15,21−24^ Many network models posit that these two times are equal, but the two processes have never been measured directly and independently on the same sample.^6,25^ Previous works have attempted to uncover this relationship, but they have been limited by the dearth of experimental techniques to measure τ_pullout_ directly, particularly in concentrated gels or bulk networks.^21,23,26−29^ The fundamental question therefore remains: what is the quantitative relationship between τ_SR_, readily measured by rheological methods, and τ_pullout_ (see Figure 1a)? By utilizing the powerful technique of time-resolved small-angle neutron scattering (TR-SANS) to determine τ_pullout_ in concentrated TPE gels, this paper provides the answer.

First applied to dilute solutions of diblock copolymers, TR-SANS involves preparing mixtures of equivalent micelles, where half the micelles contain perdeuterated core blocks and half contain normal, or hydrogenous analogs (Figure 1b). As chain exchange proceeds at a selected elevated temperature in the neutron beam, the scattering contrast decreases, resulting in a clear reduction in SANS intensity. Quantitative analysis of this decay provides direct access to τ_pullout_.^1,30−35^ More recently, TR-SANS has been used to measure chain exchange in dilute solutions of BAB triblock copolymers; however, measurements in concentrated networks have not been made previously.^22,23,36^ The key challenge to performing TR-SANS on concentrated micelle solutions is to generate an initially random mixture of h-PS core (normal polystyrene) and d-PS core (perdeuterated polystyrene) micelles. To date, the only TR-SANS measurements on more concentrated, solid-like samples were performed on a 15% solution of a body-centered-cubic (BCC)-forming diblock copolymer, where mechanical mixing (in a commercial mini-mixer with inserted ball bearings) was remarkably successful in preparing initial micelle solutions with randomly mixed isotopic cores.^37^ Such an approach is simply not feasible in the triblock case, as the network structure would necessarily be destroyed for random mixing to be achieved. Further, in both diblocks and triblocks, increasing the concentration results in even higher viscosity samples that would require increasingly aggressive mixing conditions to achieve randomly mixed cores, which could potentially impact the micellar structure and even elicit premature chain exchange.^38^

To overcome this obstacle, we employed a two-step cosolvent procedure. First, polystyrene-*b*-poly(ethylene-*alt*-propylene)-*b*-polystyrene (SEPS) micelles were prepared in very dilute solutions with the aid of a selective, volatile cosolvent (pentane), to favor the formation of flower-like micelles, *i.e*., where the PEP midblocks loop back into the same PS micellar cores. Normal (h-PS) and labeled (d-PS) micellar solutions were then mixed at low temperatures, where pullout is completely suppressed. Finally, the cosolvent was removed, leaving a concentrated solution (20% by weight) of randomly mixed micelles in the PEP-selective solvent squalane (albeit without the fully developed network structure), which could be employed for TR-SANS. Such a cosolvent procedure, which has not been employed previously for TR-SANS sample preparation, greatly expands the accessible concentration range, possibly even enabling future studies of bulk samples.

By restricting the SANS data analysis to the low wavevector (*q*) regime, we ensured that all chains must move a distance greater than the average micellar spacing, thereby eliminating concerns that the initial preponderance of midblock looping would affect the results. Unlabeled, equivalent solutions for stress-relaxation measurements were first annealed at elevated temperatures to ensure an equilibrium fraction of bridging and looping. Thus, in this work, we provide the first measurements of chain exchange in concentrated triblock networks using TR-SANS. Comparing these results to stress relaxation measurements at the same concentration quantifies the relationship between these two processes, thereby providing new molecular-level insight into the dynamics of triblock copolymer networks, and placing models of transient networks on a solid foundation.

## Results and Discussion

Two pairs of triblock copolymers with a PEP midblock and either dPS or hPS end-blocks were synthesized using sequential anionic polymerization followed by selective hydrogenation, as described previously (Table 1).^36^ The polymers have a narrow molecular weight distribution (*Đ* ≤ 1.04), and the isotopic pairs are very closely matched. One pair aimed for equivalent end-blocks in each chain, making it nominally symmetric (“SEPS-sym”), while the other deliberately introduced an asymmetric end-block structure (“SEPS-asym”). Size-exclusion chromatography (SEC) traces and proton nuclear magnetic resonance (^1^H NMR) spectra of all four polymers are included in Figures S1–S3.

**Table 1:** Summary of Polymer Characteristics

Polymer	*N*_PS,1_	*N*_PEP_	*N*_PS,2_	*x*_PS_^a^	*Đ*^b^
SEPS-sym	250	1170	250	0.30	1.03
dSEPdS-sym	235	1220	235	0.28	1.02
SEPS-asym	317	1290	221	0.29	1.03
dSEPdS-asym	307	1370	212	0.27	1.04

a Mole fraction determined using ^1^H NMR spectroscopy

b Determined using size-exclusion chromatography (SEC) with multiangle light scattering (SEC-MALS) in THF at 25 °C

**Figure 2:**
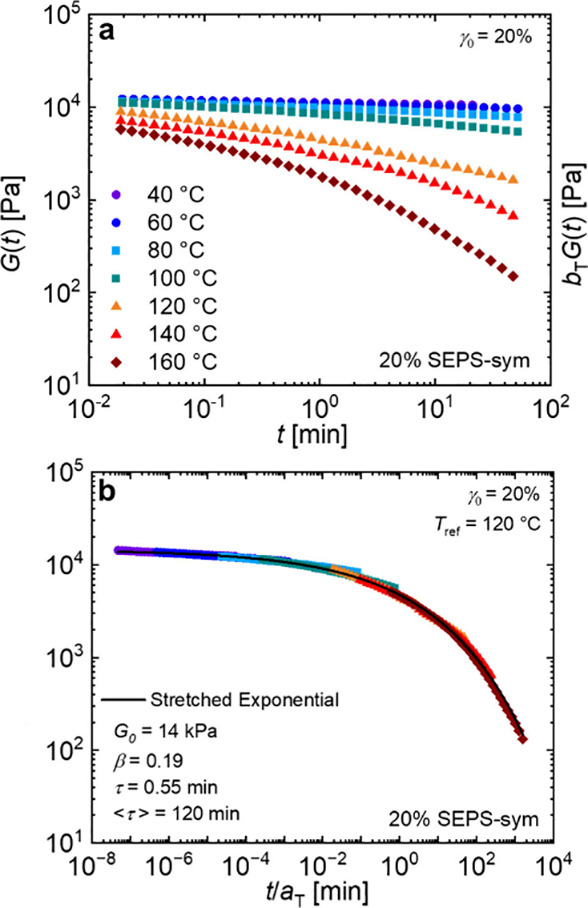
Stress relaxation measurements for 20% SEPS-sym. (a) Measurements were conducted at the indicated temperatures and then (b) shifted to generate a master curve using time–temperature superposition. Data are well-described by a stretched exponential function (solid line).

Triblock networks composed of 20 wt % PS-PEP-PS” (SEPS-sym) in squalane were annealed for 4 h at 160 °C prior to stress relaxation measurements. This annealing time, which is at least 2 orders of magnitude longer than the stress relaxation time at this temperature, was used to ensure that a network structure with a near-equilibrium fraction of loops and bridges was formed. Small-angle X-ray scattering (SAXS) indicated the formation of well-defined micelles that arranged into a disordered, liquid-like packing (Figures S4–S6). For the stress relaxation measurements, a step strain of 20% was applied, to remain within the linear viscoelastic region (see Figure S7 for strain amplitude sweeps), and the stress was monitored as a function of time for 1 h at temperatures between 160 and 40 °C. As shown in Figure 2a, stress relaxed more rapidly with increasing temperature. These curves were shifted using time-temperature superposition (*tT*s) to generate a master curve over a broad range of reduced time (Figure 2b). This method is well-established for systems above the glass transition temperature that exhibit a single family of relaxation times (e.g., homopolymer melts and solutions), but has also been successfully applied to block copolymers, including triblock networks, when the relaxation behavior is dominated by the behavior of one block.^11,14,16^ In the present case, the stress relaxation behavior is dominated by the relaxation and pullout of the PS core blocks. The empirical shift factors are consistent with those of a PS homopolymer with a suppressed *T*_*g*_ of 70 °C, determined using the Williams–Landel–Ferry (WLF) equation (Figure S8).^39^ Such a reduction in the *T*_*g*_ is expected due to a finite PEP/PS interfacial width, as described in the literature.^40−43^ For example, Lai et al. reported a *T*_*g*_ near 70 °C for a 19 kDa PS block in a styrene-isoprene diblock copolymer.^43^

The master curve is well-described by a stretched exponential (eq 1), a functional form commonly used to fit stress relaxation of disperse systems.^16,44,45^
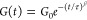
1From these fits, the mean relaxation time, *<τ>*, was estimated as 104 min at 120 °C using eq 2,
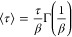
2where Γ is the gamma function.

**Figure 3:**
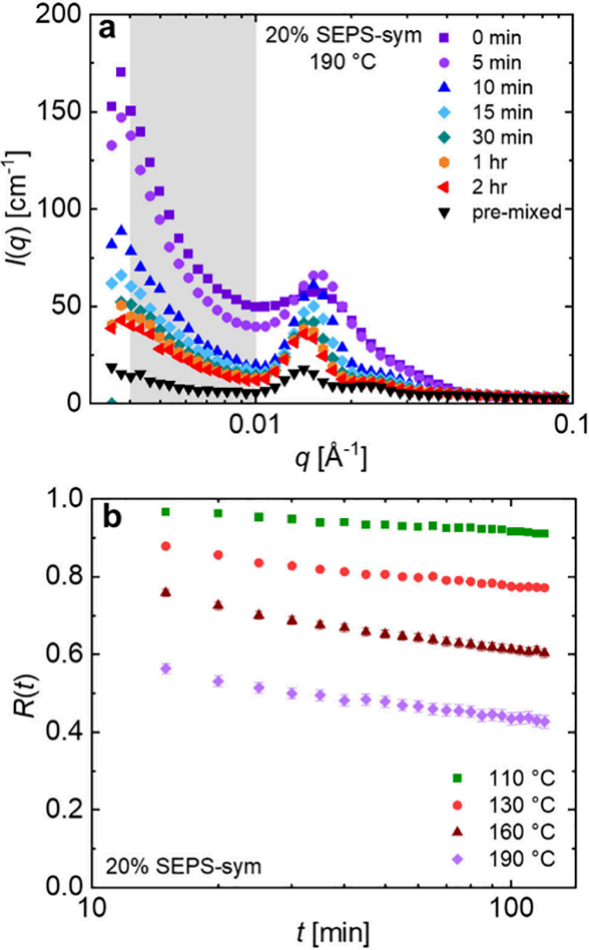
(a) TR-SANS experiments showing SANS intensity of a 20% SEPS-sym postmixed solution as a function of time following a step increase in temperature to 190 °C. Data were collected for 2 h and sliced into 5 min scans, with representative time points shown here. The low-*q* region highlighted in gray was used in the calculation of *R*(*t*) curves. (b) Normalized relaxation functions *R*(*t*) for 20% SEPS-sym obtained from TR-SANS experiments conducted on replicate postmixed samples at four different temperatures. Calculation of error bars is discussed in the SI.

To measure the dynamics of chain exchange, neutron scattering contrast was manipulated through selective deuteration of the solvent and core blocks. Postmixed solutions consisting of flower-like micelles with dPS or hPS cores within a mixed d/h-squalane (58 vol%/42 vol%) solvent were prepared using a volatile cosolvent that can be selectively evaporated (details in Materials and Methods). To measure chain exchange, postmixed samples were subjected to a step increase in temperature and neutron scattering profiles were collected every five minutes during isothermal annealing. Over time, the overall scattering intensity decreased (Figure 3a). To quantify this decrease, the normalized intensity function, *R*(*t*) was defined by
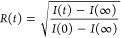
3where *I*(*t*) is the area under the scattering curve from *q* = 0.004–0.01 Å^–1^ at any given time point. Here, *I*(∞) was determined from a sample that was premixed such that every core contained approximately 50 vol% dPS and 50 vol% hPS, and *I*(0) was determined from the postmixed sample measured at room temperature prior to heating. A similar method has been employed to study chain exchange in dilute solutions of micelles and concentrated solutions of micelles that order onto a body-centered cubic lattice;^31−33,35−37^ however, the expression here is modified to integrate only over the low *q* region to restrict measurements to chains that move further than the average micellar spacing. This avoids any contributions from changes in the loop/bridge ratio and evolution of the structure factor upon heating that are evident at higher *q,* due in part to the imperfect contrast-matching arising from significant scattering from the unlabeled PEP corona blocks at these higher concentrations. Further discussion of this selection and sensitivity of the *R*(*t*) curves to the *q* range used is included in Section S5. For the 20% SEPS-sym sample, TR-SANS measurements were conducted at four different temperatures (see Figures 3b, S9, S10). As shown, increasing temperature accelerated chain exchange and thus the rate of *R*(*t*) decay.

**Figure 4:**
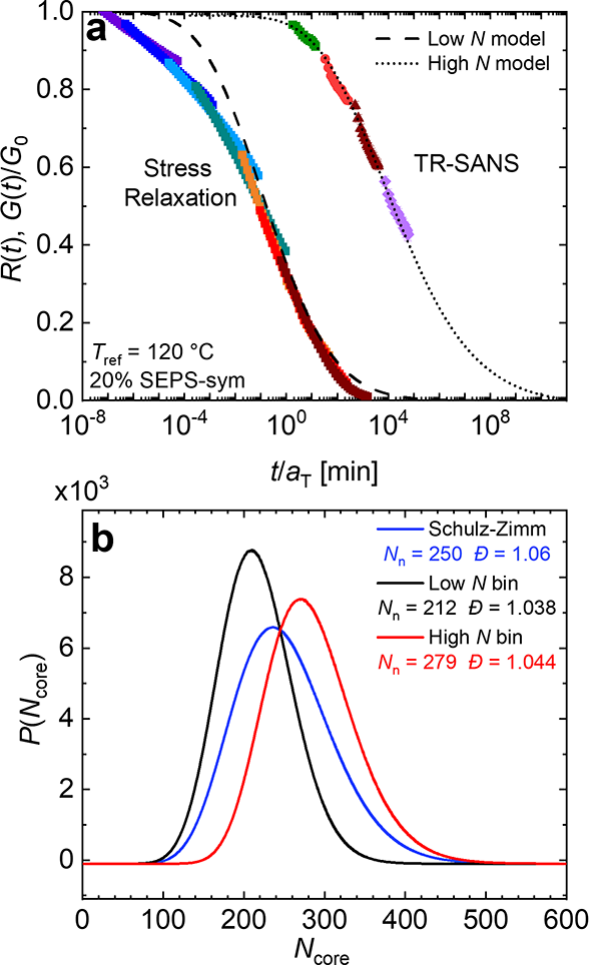
(a) Normalized stress relaxation and TR-SANS results for 20% SEPS-sym. Dashed lines represent models described in the text. (b) Overall distribution of *N*_core_ assuming a Shulz-Zimm distribution with a dispersity of 1.06 (blue) and modified distributions generated by picking pairs from the original distribution and binning them according to relative length.

For the 20% SEPS-sym samples, the *R*(*t*) curves taken at each temperature were shifted using *tT*s (Figure 4a). Notably, the shift factors for the *R*(*t*) curves were consistent with those used to shift the stress relaxation data, confirming that similar temperature-dependent processes govern both measurements. Figure 4a also shows the normalized stress relaxation behavior from Figure 2a, where *G*(*t*) is normalized to the value of *G*_0_ from the stretched exponential fit. Despite these similar shift factors between the two experiments, the time scales associated with these two measurements are different by over five orders-of-magnitude, with stress relaxing much faster than chains exchange. This remarkable and seemingly counterintuitive result will be rationalized subsequently.

Chain exchange in diblock copolymers has previously been modeled using the following expression:
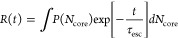
4where *P*(*N*_core_) is the distribution of core block lengths and τ_esc_ is the time scale associated with chain escape.^30−33,35,37^ Additionally, the expression can be modified to include dispersity within the corona block; however, this does not have a substantial impact on the results relative to core-block dispersity.^46^ For diblock copolymers, the time scale for chain exchange is understood to be limited by the barrier to core-block pullout and is commonly described as
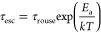
5where τ_rouse_ is the longest relaxation (Rouse) time associated with the unentangled bulk polystyrene core and the activation energy for chain pullout, *E*_a_, is defined as
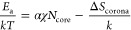
6where *k* is Boltzmann’s constant, *T* is temperature, α is a constant of order unity, χ is the interaction parameter between the core block and the corona/solvent matrix, and Δ*S*_corona_ is an additional term to account for any changes in the stretching entropy of the corona chain upon pullout.^31−33,37,46^ For the triblock polymers used in this study, the rate of chain exchange is expected to be dictated by core block expulsion, with this process being very slow relative to diffusion between micelles. The time scale for diffusion between micelles is estimated to be on the order of 10^–2^ s based on the reptation time of PEP in squalane and the intermicelle distance of SEPS (details in Section S7).^47,48^

As noted in the introduction, for a bridged network of triblock copolymers, the canonical assumption is that the rate of end-block pullout dictates stress relaxation, hence the time scale of chain exchange for a triblock should be similar to that for stress relaxation. In contrast, the data presented in Figure 4a show for the first time that stress relaxation is many orders-of-magnitude faster than triblock exchange measured on the same sample. Previously, a qualitatively similar conclusion was inferred based on comparisons of concentrated triblock network stress relaxation with dilute solution diblock chain exchange.^15^ At face value, this large difference is hard to reconcile; how can a sample flow on a time scale much shorter than chains can exchange? However, there is a straightforward explanation. These differences can be rationalized based on a slight asymmetry in the end-block lengths, even for low-dispersity, nominally symmetric triblock copolymers.^15^ In every triblock, statistically there will be one end-block that is slightly shorter than the other. The linear dependence of *E*_a_ on *N*_core_, coupled with the “exponential of an exponential” dependence of *R*(*t*) on *N*_core_ implied by eqs 4–6, leads to a hypersensitivity to core-block dispersity (*Đ*_core_). Stress relaxation of an elastically effective strand, i.e., a bridged PEP midblock, only requires one PS end-block to escape, whereas chain exchange measured by TR-SANS requires both end-blocks to eventually escape. To account for this, revised *P*(*N*_core_) distributions were generated by randomly sampling two chains from a low-dispersity, Shulz-Zimm distribution of *N*_core_ with the same overall dispersity as the triblocks. The lengths of each pair of end-block chains were compared and the smaller was placed in a “small *N*” bin and the larger was placed in a “large *N*” bin. This process was repeated for 10^6^ pairs of chains to generate two new distributions *P*(*N*_core,S_) and *P*(*N*_core,L_), which contain the chains in the small *N* and large *N* bins, respectively. For an original distribution with *Đ*_core_ = 1.06, this process led to substantial differences in the number-average core length (*N*_n_) for each of the three distributions, as shown in Figure 4b. This value of *Đ*_core_ = 1.06 is similar to that determined using SEC-MALS on the PS blocks recovered following a cross-metathesis degradation of the PI block (Figure S2).^49^ Modest differences between these values are likely due to a combination of blending of dPS- and hPS-containing polymers and limitations of SEC-MALS. The sensitivity of this model to *Đ*_core_ is illustrated in Figure S19.

In addition to the impact of core block distribution, the triblock architecture also enables two different types of chain configurations: loops and bridges. The bridged chains are expected to experience a greater degree of stretching than loop chains and contribute as elastically effective strands in the network.^6,23,50−52^ Consequently, the entropy penalty associated with a bridge relative to the unstretched state is defined as
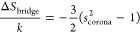
7where *s*_corona_ is a corona stretching parameter defined as *h*/⟨*h*⟩_0_, where *h* is the end-to-end distance of a bridged corona chain and ⟨*h*^2^⟩_0_ is the mean-squared end-to-end distance of a corona chain if it were to obey Gaussian statistics, i.e., ⟨*h*^2^⟩_0_ = *N*_corona_*b*(2), where *b* is the statistical segment length of PEP (8.0 Å).^48,50,51^ Estimates of *s*_corona_ for 20% SEPS-sym from SAXS data and its associated sensitivity are discussed in Section S8. Resultingly, the change in corona entropy upon bridge breakage is
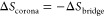
8

Using these models, the combined stress relaxation and TR-SANS data in Figure 4a can be fully described using eqs 4-7 with a single value of *αχ* = 0.07, and employing the appropriate distribution function (*P*(*N*_core,S_) versus *P*(*N*_core,L_)) and the relevant entropic contribution associated with relief of bridge chain stretching. The result is shown as the dashed curves marked “low *N* model” and “high *N* model” in Figure 4a. This agreement with both stress relaxation and TR-SANS is excellent. Here we note that the “low *N* model” deviates from the stress relaxation data at the shortest times, but this is understandable in that the stress relaxation function has some faster relaxing contributions that are not related to chain exchange. The value of *αχ* obtained is also satisfyingly close to previously reported values of χ for PS and PEP, suggesting that α is near unity in this case, though the exact value of χ(*T*) is difficult to measure.^37,53^

For 20% SEPS-sym, the stress relaxation data can be described by the enthalpic term associated with pullout of the shorter *N*_core,S_ block and a reduction in activation energy that could reasonably be associated with the entropic gain of a bridge breaking during the stress relaxation process (eq 7). This indicates that only one core-block chain (the smaller one) is required to pullout for stress relaxation to occur, *i.e., τ*_pullout,S_ = τ_SR_. In contrast, while the shorter block can pullout and bridge to a neighboring micelle, and move dynamically between a loop and bridge configuration among nearest neighboring micelles, full chain exchange cannot occur until the longer block also pulls out and exchanges, and thus *P*(*N*_core,L_) dictates the rate-limiting step of triblock chain exchange measured using TR-SANS, *i.e. τ*_pullout,S_ ≪ τ_pullout,L_ = τ_chain exchange_.

**Figure 5:**
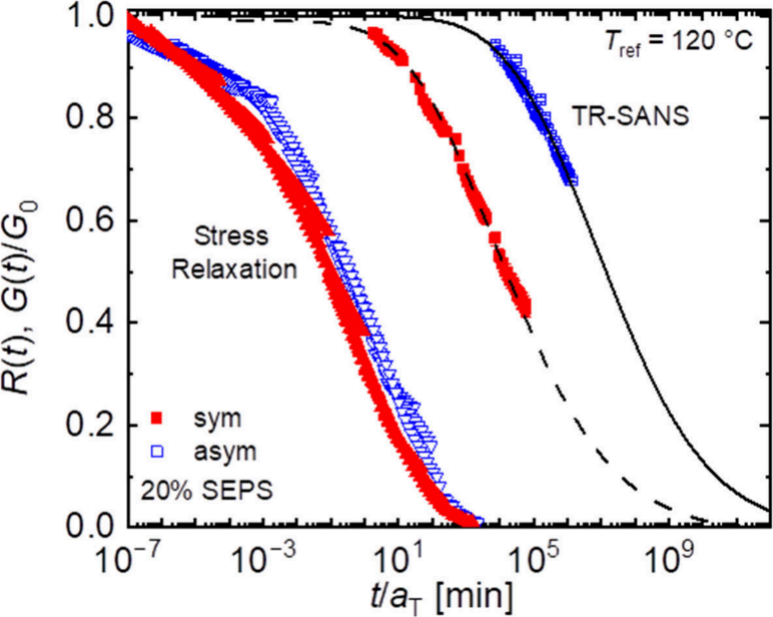
Normalized stress relaxation and TR-SANS decay functions for 20% SEPS-sym and SEPS-asym formulations. Lines represent models for the TR-SANS data where for both cases.

To further test this key hypothesis, an additional pair of intentionally asymmetric triblocks (SEPS-asym) were synthesized. These triblocks have a similar shorter block distribution to *P*(*N*_core,S_), but a higher long block distribution, i.e., the overall degree of asymmetry is higher. TR-SANS measurements were also conducted at 20%, and the resulting SANS traces and *R*(*t*) curves are included in Figures S11 – S13. As shown in Figure 5, stress relaxation occurs on a very similar time scale for both polymers, as expected, but chain exchange is much slower for SEPS-asym than for SEPS-sym. This slower chain exchange rate can be fully accounted for by the higher *N*_core,L_ values for SEPS-asym. Using this new *P*(*N*_core,L_) distribution, the data is well-described using eqs 4-6 with the same value of *αχ* = 0.07. This result provides compelling evidence for the interpretation proposed here.

These results are consistent with the findings of Peters and Lodge,^15^ who studied stress relaxation in similar PS-PEP-PS triblock networks, but appear to differ from those of Zinn et al.^23^ who found that the rate of chain exchange in dilute difunctional telechelic polymers was similar to that of stress relaxation at higher concentrations. In that work, the core blocks were composed of C_22_ alkane chains with no end-block dispersity, as is present in triblock copolymers such as those studied here. Consequently, the rate of pullout for both of the end-blocks would be identical and thus stress relaxation and chain exchange rate would be expected to be comparable at the same concentration. These results reconcile the current data with both of these previous studies.

These results also support the “walking diffusion” mechanism first hypothesized by Yokoyama and Kramer,^50^ where τ_esc_ ∼ exp(*αχN*_core_) in percolated networks. This mechanism proposes that in concentrated systems, end-blocks can pullout sequentially, with the first terminal block inserting into a neighboring micelle and forming a bridge prior to the second block pulling out.^15,22,23,50^ This scaling contrasts with that of the “double activation” mechanism, hypothesized to occur in dilute triblock solutions, where both core blocks within a given chain must pullout at the same time for a chain to escape and move to a new micelle, resulting in a τ_esc_ ∼ exp(2*αχN*_core_) scaling.^50^

This work brings new molecular-level insight into the relationship between stress relaxation and chain exchange in triblock copolymer networks. These findings are translatable to the billion-dollar TPE industry, where triblocks networks are often used both with (e.g., hot melt adhesives) and without aliphatic plasticizers (for which squalane is a reasonable proxy). These results show unequivocally that PS end block escape from the core governs both chain exchange and stress relaxation. Squalane is highly selective for the PEP domain resulting in essentially no solvent in the PS core. As has been shown in detail previously, if a block copolymer solution is more than *ca*. 50 °C below the critical micelle temperature (CMT), there is negligible solvent in the core.^57^ Based on previous work on PS-PEP in squalane, we estimate the CMT to be much greater than 200 °C.^58^ Therefore, squalane does not alter the dynamics of the PS blocks, a conclusion also supported by the successful use of the Rouse time for bulk PS in eq 5.

## Conclusions

In this work, the first measurements of chain exchange in concentrated triblock networks were conducted using TR-SANS. A new method using a selective cosolvent was employed to prepare concentrated triblock solutions with randomly mixed isotopically labeled cores for TR-SANS measurements. These experiments revealed that macroscopic stress relaxation occurs many orders-of-magnitude faster than chain exchange when measured on the same sample at the same concentration. Satisfyingly, this difference can be quantitatively explained by accounting for asymmetries in the core block lengths, both within nominally symmetric and intentionally asymmetric triblock copolymers. Specifically, stress relaxation only requires pullout of the shorter end-block, whereas chain exchange requires escape of both end-blocks. Nominally symmetric triblock copolymers with a low degree of core-block dispersity exhibit more than a five-orders-of-magnitude difference in these two experimental rates, while the intentionally asymmetric triblock has an eight-orders-of-magnitude difference. These results confirm that macroscopic stress relaxation and molecular-level chain exchange are dictated by the same underlying process of single-chain pullout, as has been widely assumed, but macroscopically stress relaxation occurs more rapidly than chain exchange, as only the shorter block must pullout for stress relaxation while the longer block pullout limits chain exchange rate. This conclusion is relevant to many types of soft materials that rely on thermoreversible nanoscale molecular self-assembly to create favorable physical properties dictated by macromolecular dynamics.^54−56^

## Materials and Methods

### Polymer Synthesis

Polymers were synthesized using sequential anionic polymerization of styrene, isoprene, and styrene, followed by selective hydrogenation of the polyisoprene block. These reactions require the use of pyrophoric materials. Care was taken to ensure that an air-free environment was maintained throughout the reaction and that all materials were appropriately quenched after use. Adequate training and a thorough assessment of safety hazards and equipment were performed prior to conducting reactions. Styrene and perdeuterated styrene-*d*_8_ were purified by stirring for 12 h over calcium hydride, followed by freeze–pump–thaw cycles, vacuum distillation, and stirring twice over butylethylmagnesium at 40 °C for 1 h. Isoprene was purified through freeze–pump–thaw cycles, vacuum distillation, and stirring twice over *n*-butyl lithium in an ice bath for 30 min. The polymerizations were conducted in dry cyclohexane at 40 °C under 5 psi of argon. The *sec*-butyllithium initiator (1.4 M in hexanes) was allowed to mix with cyclohexane for 1 h prior to addition of purified styrene monomer. After more than 12 h, an aliquot of the reaction was taken through a rubber septum using a gastight syringe and terminated into methanol sparged with argon, for characterization. The purified isoprene was then added to the reactor and stirred for 6 h. Finally, additional purified styrene monomer was added and allowed to react for at least 12 h. The reaction was terminated with degassed methanol. The polymer was precipitated in methanol with butylated hydroxytoluene (BHT) to prevent degradation, and dried under vacuum.

The polyisoprene block was selectively hydrogenated using a nickel/aluminum catalyst at 77 °C and 500 psi H_2_ for 20 h. The preparation of the catalyst required the use of pyrophoric materials and the reaction was run under high pressure hydrogen, which is highly flammable. Care was taken to maintain an air-free environment during catalyst preparation and the reactor was equipped with a pressure relief valve to prevent overpressurization. Prior to hydrogenation, the polymer was dissolved in benzene, filtered through basic alumina, and freeze-dried. In an environment free of air and water, the polymer was redissolved in dry cyclohexane and cannula transferred into a 1 L stainless steel Parr reactor under 5 psi of argon. The catalyst was prepared by mixing 20 mL of 0.1 M nickel(II) 2-ethyl hexanoate in cyclohexane with 6 mL of 1 M triethylaluminum in hexanes in an ice water bath. The catalyst solution was transferred using a cannula to the Parr reactor, followed by the addition of H_2,_ heating to 77 °C, and stirring for the course of the reaction. Saturation of the polyisoprene double bonds was monitored using ^1^H NMR spectroscopy. After completion, the catalyst was deactivated by stirring with 8 wt % citric acid in distilled water for 24 h; the solution turned from dark brown to white during this step. The top organic layer was extracted, filtered through basic alumina, precipitated in methanol, and dried under vacuum to recover the final polymer.

### Micelle Solution Preparation

All solutions were prepared to a concentration of 20 wt % polymer in squalane. For TR-SANS measurements, where dSEPdS polymers were used, concentrations were instead calculated on a volume basis (18 vol%) to account for the higher repeat unit molar mass of dPS.^34^ Triblock copolymers were dissolved at 1 vol% in benzene (a good solvent for both blocks) and freeze-dried. Micelle solutions were then formed using a cosolvent method, where polymers were dispersed in squalane and a cosolvent of 95 vol% pentane and 5 vol% dichloromethane to form a dilute (1 vol%) solution, to favor formation of flower-like micelles. The cosolvent was then evaporated to yield a polymer solution in squalane at the target concentration. For stress relaxation measurements, evaporation was conducted under ambient conditions and then samples were annealed under nitrogen gas at 160 °C for 4 h to promote micelle bridge formation. For TR-SANS measurements, where bridge formation was to be minimized, cosolvent solutions were placed in a dry ice/ethylene glycol bath (−10.5 °C) or dry ice/acetone bath (−77 °C) and the cosolvent was removed using dynamic vacuum. For TR-SANS measurements, the flower-like micelles were then rediluted to 3 vol% using the cosolvent mixture prior to mixing of the dSEPdS and SEPS solutions at reduced temperature and subsequent removal of the cosolvent using dynamic vacuum at subambient conditions. Dynamic light scattering (DLS) on diluted solutions was used to confirm that this preparation procedure did not induce significant bridge formation and that postmixed samples were therefore composed of flower-like micelles (Figures S15 and S16).

### Stress Relaxation

Stress relaxation measurements were conducted on an ARES-G2 rheometer using a 25 mm parallel plate geometry at temperatures between 40 and 160 °C. All samples were annealed at 160 °C prior to measurement. A step strain of 20% was applied and the stress as a function of time was recorded for 1 h. All measurements were conducted under a N_2_ gas flow and allowed to equilibrate at each new temperature for 10 min prior to data collection.

### Small-Angle X-ray Scattering

Small-angle X-ray scattering (SAXS) measurements were conducted on a lab-source Xenocs Ganesha instrument and at Sector 11-BM of the National Synchrotron Light Source – II (NSLS-II) at Brookhaven National Laboratory (BNL). Both instruments were calibrated using silver behenate. Measurements on the Ganesha instrument were performed under vacuum using a Genix 3D Cu microfocus X-ray tube (λ = 1.54 Å) with a Dectris Eiger R 1 M detector. A sample-to-detector distance of 1.05 m and an exposure time of 5 min yielded SAXS data over the range of 0.005 < *q* < 0.12 Å^–1^ where *q* = (4π*/λ*)sin(θ/2). Synchrotron measurements (λ = 0.918 Å) employed a Pilatus 2 M detector, a sample-to-detector distance of 5.02 m, and an exposure time of 3 s yielding SAXS data over 0.003 < *q* < 0.18 Å^–1^. Isotropic two-dimensional patterns were azimuthally integrated using beamline software or Datasqueeze to generate 1D scattering profiles of intensity *I*(*q*) versus *q*.^59^

### Time-Resolved Small-Angle Neutron Scattering

Small-angle neutron scattering (SANS) measurements were conducted at the CG2 GP-SANS beamline at the High Flux Isotope Reactor at Oak Ridge National Laboratory (ORNL). These experiments used λ = 4.75 Å, an 8 mm diameter beam, a sample-to-detector distance of 15 m, a 1 m^2^ area detector with 8 × 4 mm^2^ pixel resolution, and an off-center beamstop to yield data over 0.0035 < *q* < 0.1 Å^–1^. Select measurements at lower temperatures for 20% SEPS-sym samples were measured at a 7 m sample-to-detector distance with a centered beamstop, resulting in the range 0.007 < *q* < 0.5 Å^–1^. The detector was then moved to the 15 m sample-to-detector distance described above to maximize the low-*q* region for *R*(*t*) analysis. Absolute intensity was calibrated using a Porasil standard.

Ambient measurements used an exposure time of 5 min. For all measurements, samples were loaded between quartz plates (Esco Optics, 1” diameter, 0.063” thickness) and separated by an aluminum spacer (McMaster Carr, 0.5” I.D., 0.75” O.D., 0.032” thickness). Samples were sealed using a high-temperature sealant, and loaded into Ti blocks housed within a furnace. Postmixed samples (*t* = 0) for time-resolved SANS (TR-SANS) measurements were prepared using the cold-evaporation method detailed above. Premixed micelles (*t* = ∞) were prepared by freeze-drying both the dSEPdS and SEPS triblock copolymers together from benzene, redissolving within the cosolvent mixture with squalane, evaporating at room temperature, and annealing under N_2_ at 160 °C (Figure 1). For TR-SANS measurements, the furnace was preheated to the target temperature and the postmixed sample was loaded. After loading, the first five minutes of time was neglected to allow for sample thermal equilibration. TR-SANS measurements were conducted isothermally for 2–3 h and data were time-sliced and averaged over every 5 min.

## Supporting Information Available

Size-exclusion chromatography and ^1^H NMR spectroscopy of triblock copolymers, SAXS traces and fits, amplitude sweeps of triblock networks and shift factors used to generate master curves, TR-SANS data for all temperatures and samples, *R*(*t*) error and sensitivity analysis, dynamic light scattering and discussion of micelle preparation, rheology of PEP homopolymer in solution and estimation of diffusion time, stress relaxation data for SEPS-asym (PDF)
