# Supplementary material for: How Does the Rate of Chain Exchange Relate to Stress Relaxation in Triblock Copolymer Networks?
Source: ACS Cent Sci. 2025 Feb 20;11(3):422–30. doi: 10.1021/acscentsci.4c02031 (PMC11950850; doi:10.1021/acscentsci.4c02031)
Supplement: Supplementary file 1 — oc4c02031_si_001.pdf [file oc4c02031_si_001.pdf]

Supplemental Information

for

"How does the rate of chain exchange relate to stress relaxation in triblock copolymer networks?"

Joanna M. White, Taehyoung Kim, Frank S. Bates, Timothy P. Lodge

S1. Polymer characterization

Size-exclusion chromatography (SEC) measurements were conducted on an Agilent Infinity 1260 HPLC system with three Waters Styragel HR columns. A Wyatt HELEOS-II multiangle light scattering detector and a Wyatt Optilab T-rEX differential refractive index detector were used. Samples were prepared in 2 mg/mL solutions in THF and filtered through a 0.22-µm PTFE filter prior to measurements. All polymers had a narrow dispersity. The traces indicate the presence of small fractions of PS homopolymer and coupled triblock that are not anticipated to dramatically impact the micelle dynamics; these were not included in the molar mass and dispersity calculations.

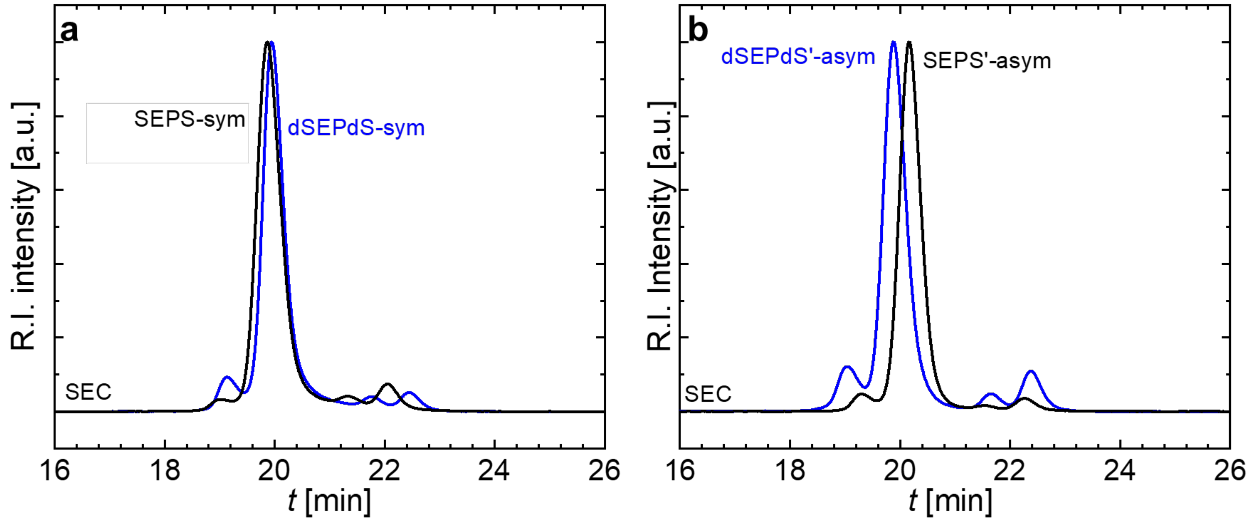

Figure S1. SEC traces of (a) SEPS-sym and dSEPdS-sym and (b) SEPS-asym and dSEPdS-asym

Aliquots were taken of the first PS block during synthesis to characterize the molar mass and first block distribution. A gastight syringe was used to sample the reaction solution and the aliquot was precipitated in methanol that had been sparged with Ar. To characterize the overall core-block distributions, a cross-metathesis degradation procedure of the 1,4-PI block in the PS-PI-PS triblock was used. This procedure was previously developed and employed to degrade 1,4-polybutadiene, but was able to be extended to degrade the PI block by employing slightly harsher reaction conditions.[1] In brief, 150 mg (1 µmol) of PS-PI-PS mixed with 5 mg (6 µmol) of Grubbs 2nd generation catalyst. The mixture was placed in a round bottom flask equipped with a rubber septum and sparged with Ar for 10 min. Separately, a 1:1 (vol) mixture of benzene and 1-hexene was sparged with Ar for 10 min and a gastight syringe was used to transfer 4 mL of the solvent

mixture into the flask with the polymer and catalyst (0.02 mol 1-hexene). The polymer was dissolved by stirring and the reaction was stirred at 40 °C for >12 h prior to quenching with 10 μL (100 μmol) of ethyl vinyl ether. The degraded product was isolated through three sequential precipitations in methanol and redissolutions in cyclohexane.

SEC traces of the aliquot and the degraded product for SIS-sym show very similar distributions, confirming that the polymer is symmetric. In contrast, the degraded product of SIS-asym has a lower molar mass distribution than that of the aliquot taken after the synthesis of the higher molar mass block. Comparing the two degraded products shows that the low molar mass (high elution time) region is similar, but the asymmetric polymer has a higher molar mass shoulder due to the presence of the larger PS block (Figure S2).

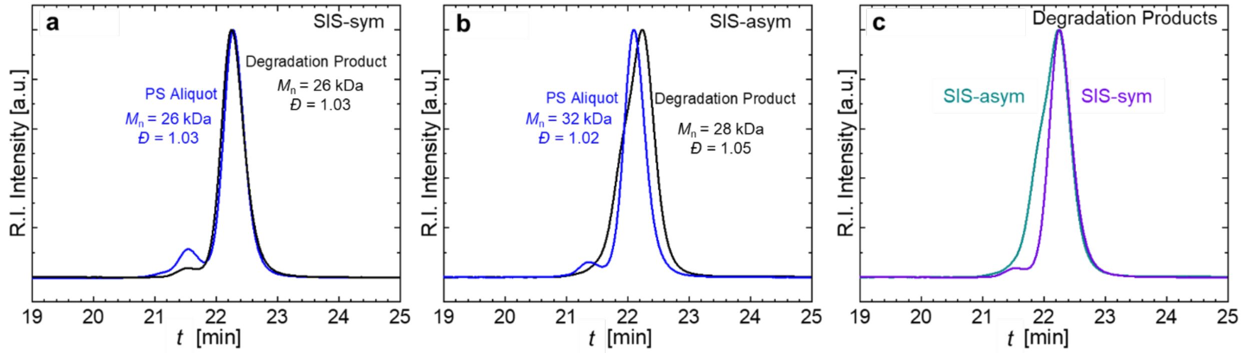

Figure S2. SEC traces of PS aliquot and degradation product from (a) SIS-sym and (b) SIS-asym. (c) Comparison of degradation products for SIS-sym and SIS-asym.

Representative ^{1}H NMR results for SEPS-sym during each step of the synthesis process are shown in Figure S3.

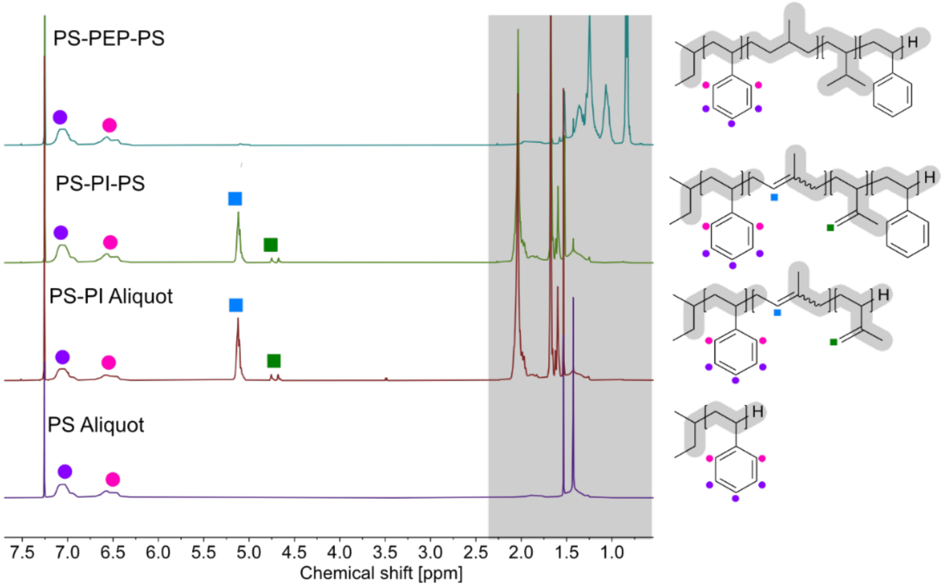

Figure S3. Representative ^{1}H NMR spectra for the SEPS-sym triblock polymer at each step of the synthesis process

S2. Small-angle X-ray scattering (SAXS)

SAXS was used to probe the structure of triblock solutions used for stress relaxation measurements. Prior to measurements, samples were annealed at 160 °C for various amounts of time (Figure S4). As shown, the peaks and form factor minimums sharpened after 1 h and 4 h of annealing, but the structure remained fairly unchanged at longer annealing times, indicating that 4 h of annealing was long enough to ensure no significant structural evolution during the stress relaxation measurements.

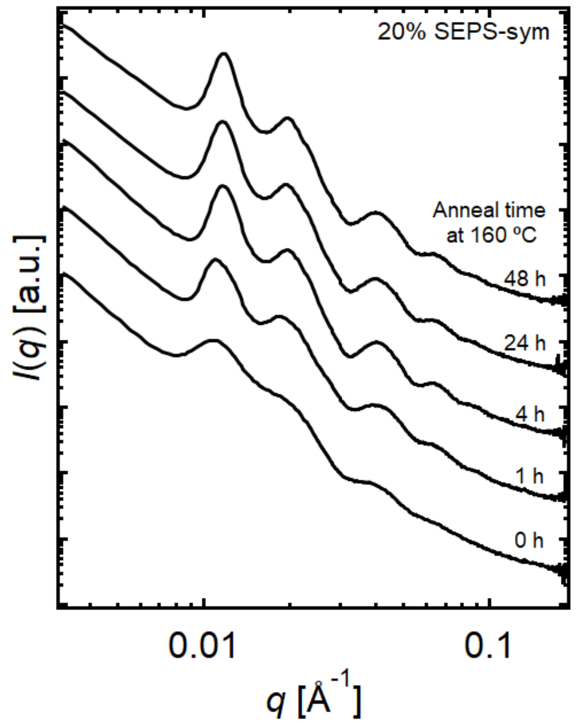

Figure S4. SAXS traces of 20% SEPS-sym annealed for various amounts of time at 160 °C.

SAXS traces of each formulation used for TR-SANS were fit to a spherical form factor with a hard sphere structure factor. These fits were performed using the Sasview package.[2] For SAXS measurements, all core compositions share a uniform electron density and therefore the scattering intensity $I(q)$ follows eqn. S1.

$$I(q) \sim (\Delta\rho)^2 P(q) S(q) \tag{S1}$$

where $\Delta\rho$ is the difference in electron density between the core and matrix, $P(q)$ is the form factor and $S(q)$ is the structure factor. To ensure convergence of fits, the following fitting procedure was used. First, the higher q regions (beyond the inflection point after the first peak, and highlighted in purple in Figure S5), were fit to a spherical form factor. In this high q region, the structure factor approaches one, and therefore only form factor contributions are important. For these fits, the electron density of the core was set to that of PS, the electron density of the solvent/matrix was set to that of squalane, and the core radius, R_{core}, dispersity of the core, and a scale factor (Φ) were varied. These values were then held constant and the q range was expanded to include the lower q

region where structure factor contributions are significant. A second fit was performed across this full region while allowing the hard-sphere radius, R_{HS}, to vary. Further details on these models are included in the following references.[2-4]

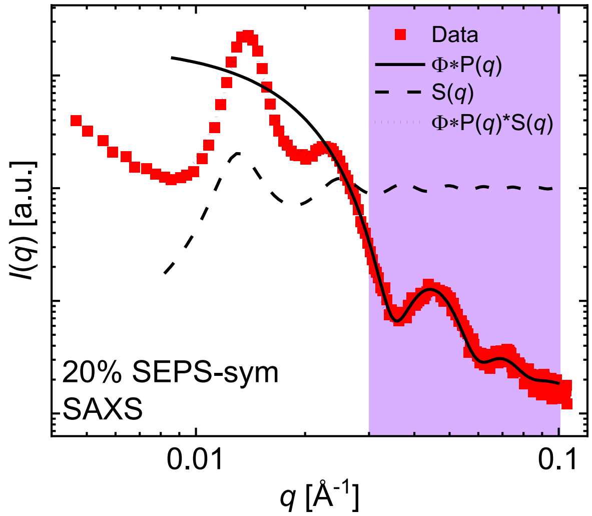

Figure S5. Example of the fitting process. First the high q region (denoted in purple) was fit to the spherical form factor (solid line). Then, the fit was expanded to include the structure factor and the full q range.

These fits show that R_{core} and R_{HS} are similar across the d-core (dSEPdS only), h-core (SEPS only), post-mixed (prior to TR-SANS measurements), and pre-mixed samples (Figure S6). They display only minor differences, which are likely due to slight molar mass mismatches between the polymers and, for the post-mixed sample, the inevitable absence of thermal annealing.

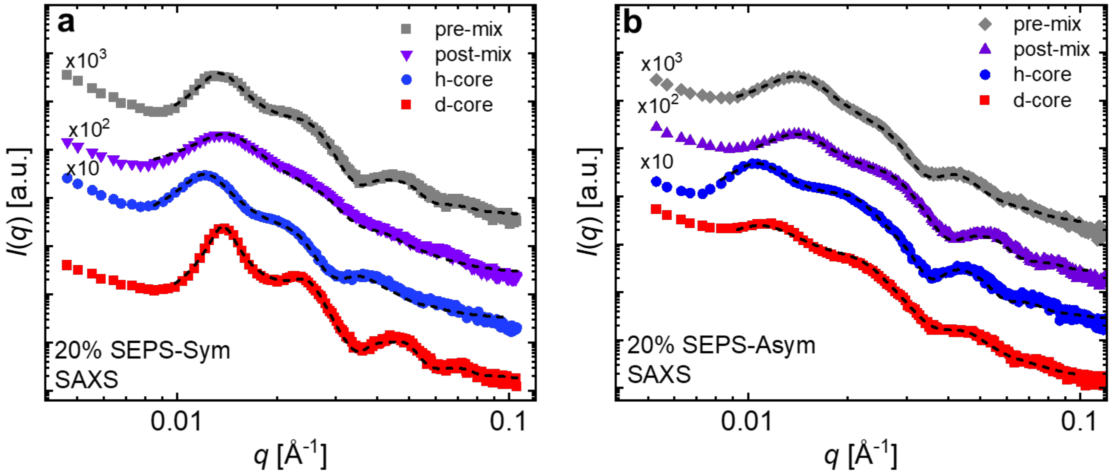

Figure S6. SAXS traces of (a) 20% SEPS-sym formulations and (b) 20% SEPS-asym formulations with different core blocks. Here, pre-mixed contains 50%/50% d-PS and h-PS and post-mixed contains

segregated cores of pure d-PS and h-PS. Dashed lines represent fits to a spherical form factor with a hard sphere structure factor.

Table S1. Fitting results from SAXS traces shown in Figure S5.

Formulation	R_{core} [Å]	R_{HS} [Å]
**SEPS-sym: d-core**	125	253
**SEPS-sym: h-core**	130	253
**SEPS-sym: post-mix**	112	218
**SEPS-sym: pre-mix**	122	244
**SEPS-asym: d-core**	117	274
**SEPS-asym: h-core**	124	295
**SEPS-asym: post-mix**	107	219
**SEPS-asym: pre-mix**	124	216

S3. Linear Viscoelastic (LVE) determination and shift factors

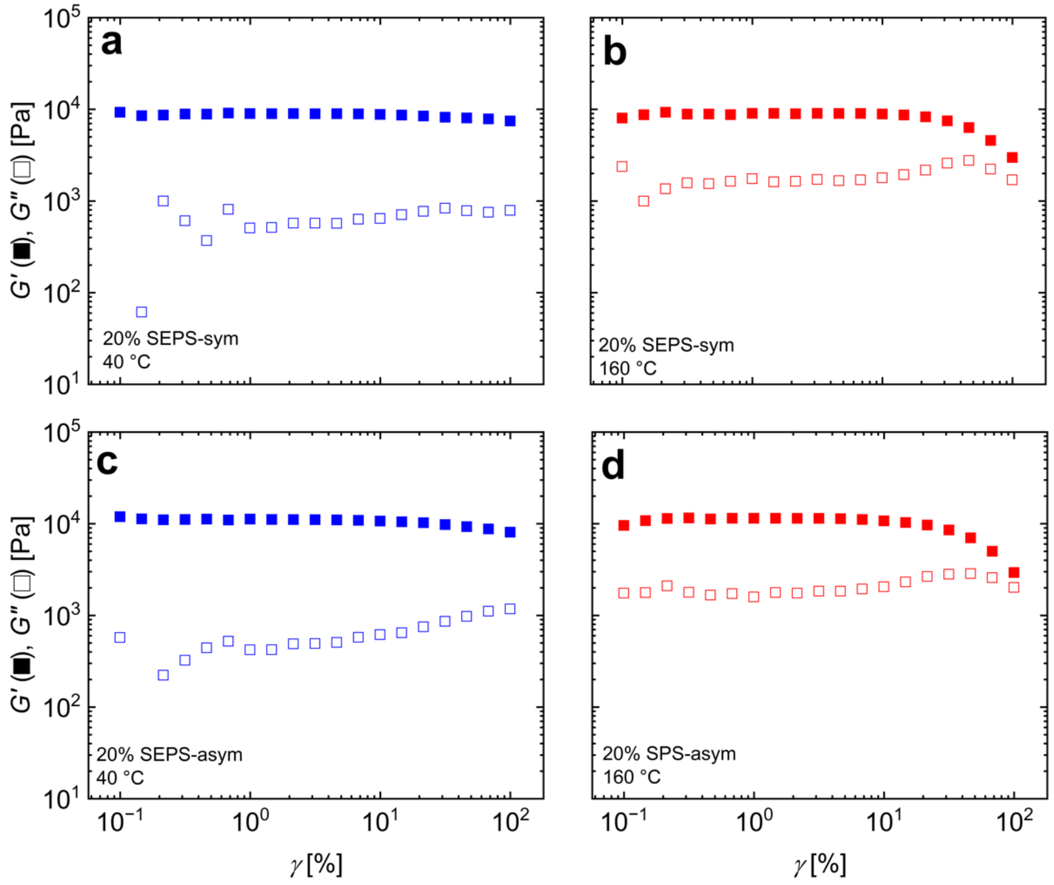

Figure S7. Amplitude sweeps conducted at ω = 1 rad/s for (a) SEPS-sym at 40 °C, (b) SEPS-sym at 160 °C, (c) SEPS-asym at 40 °C, and (d) SEPS-asym at 160 °C.

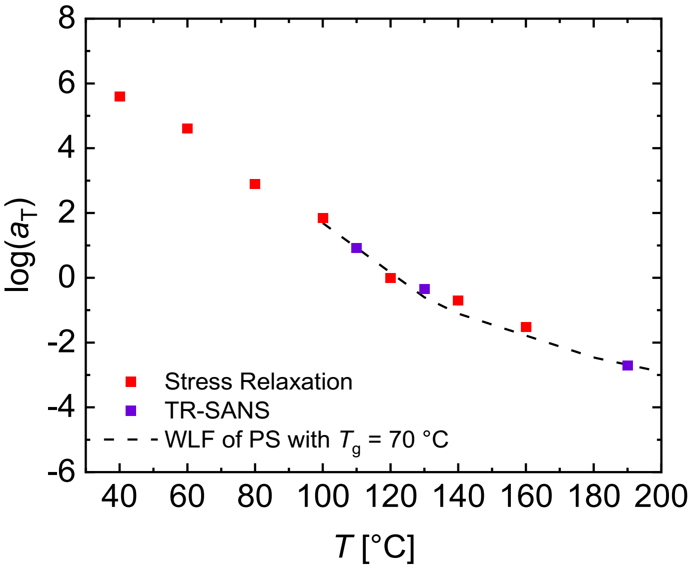

Figure S8. Shift factors determined empirically for stress relaxation data and TR-SANS data for 20% SEPS-sym. The dashed line represents the shift factors of PS homopolymer, which obey a WLF relationship, when the T_g is suppressed to 70 °C.

S4. Time-resolved small-angle neutron scattering data

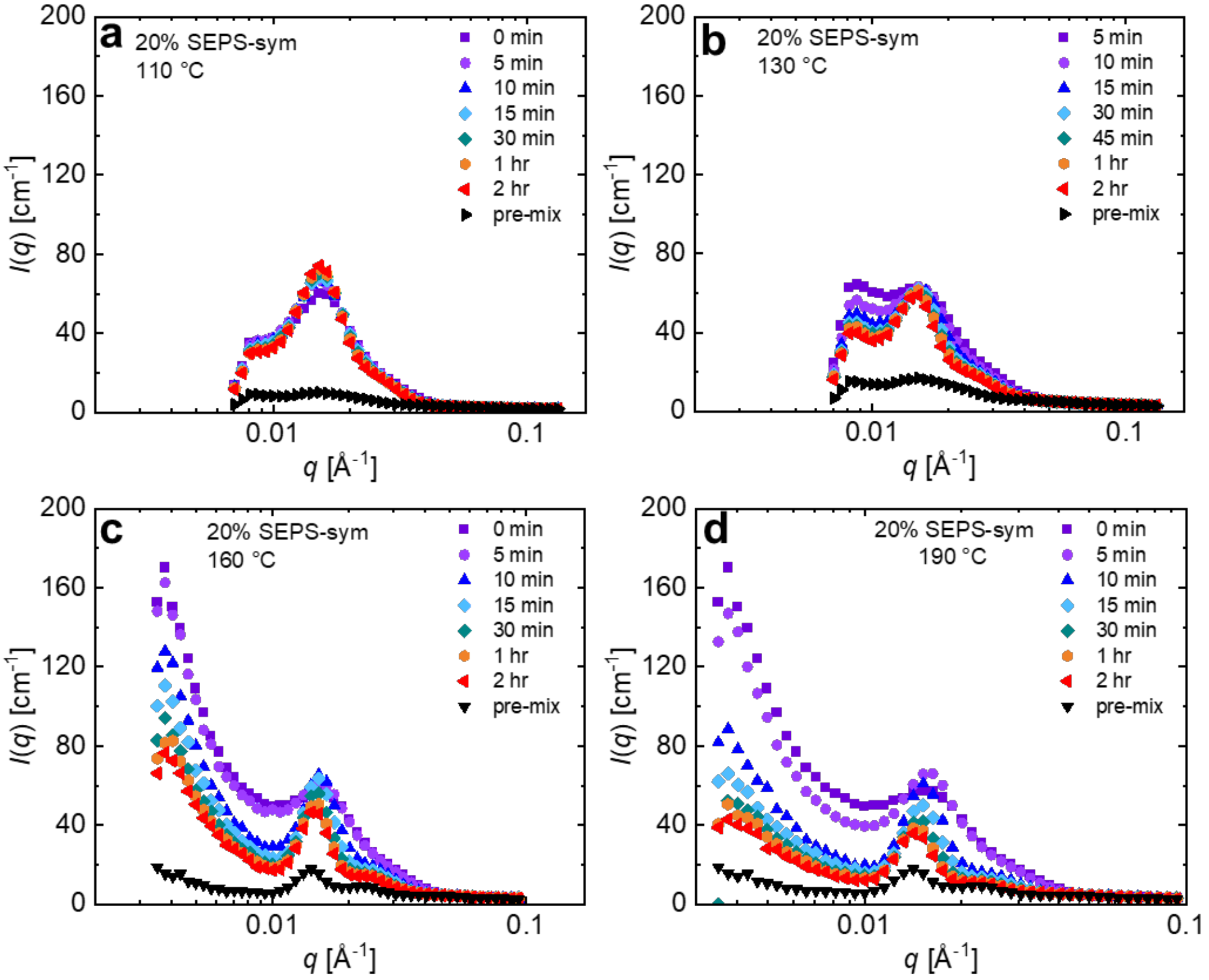

Figure S9. 1D TR-SANS traces for 20% SEPS-sym at four different temperatures. Each temperature used a fresh, replicate post-mixed sample. The lower two temperatures were run with a 7 m sample-to-detector distance, while the higher two temperatures were run with a 15 m sample-to-detector distance to increase the accessible low-q range used for the $R(t)$ analysis.

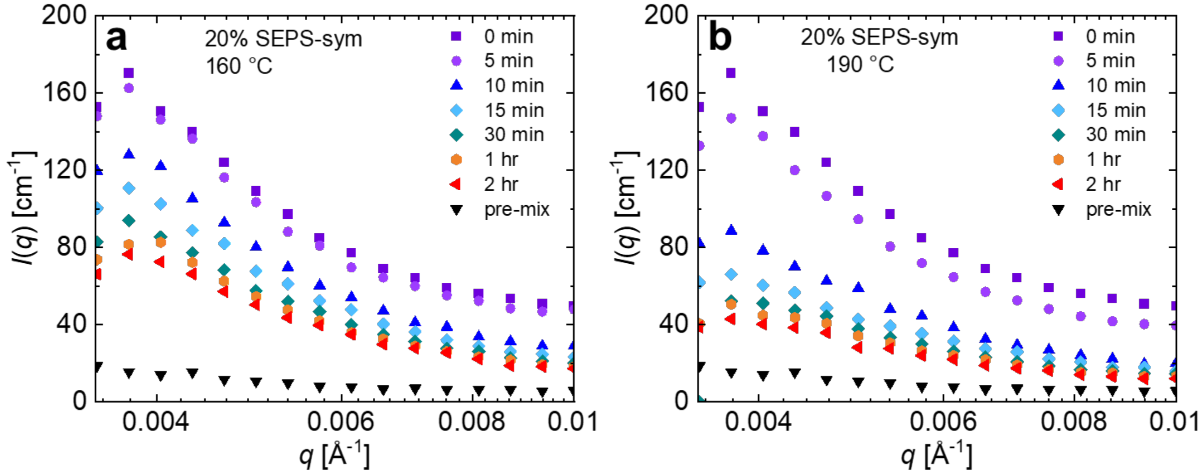

Figure S10. 1D TR-SANS traces for 20% SEPS-sym shown in Figure S9, zoomed in on the *q*-region used for *R*(*t*) analysis.

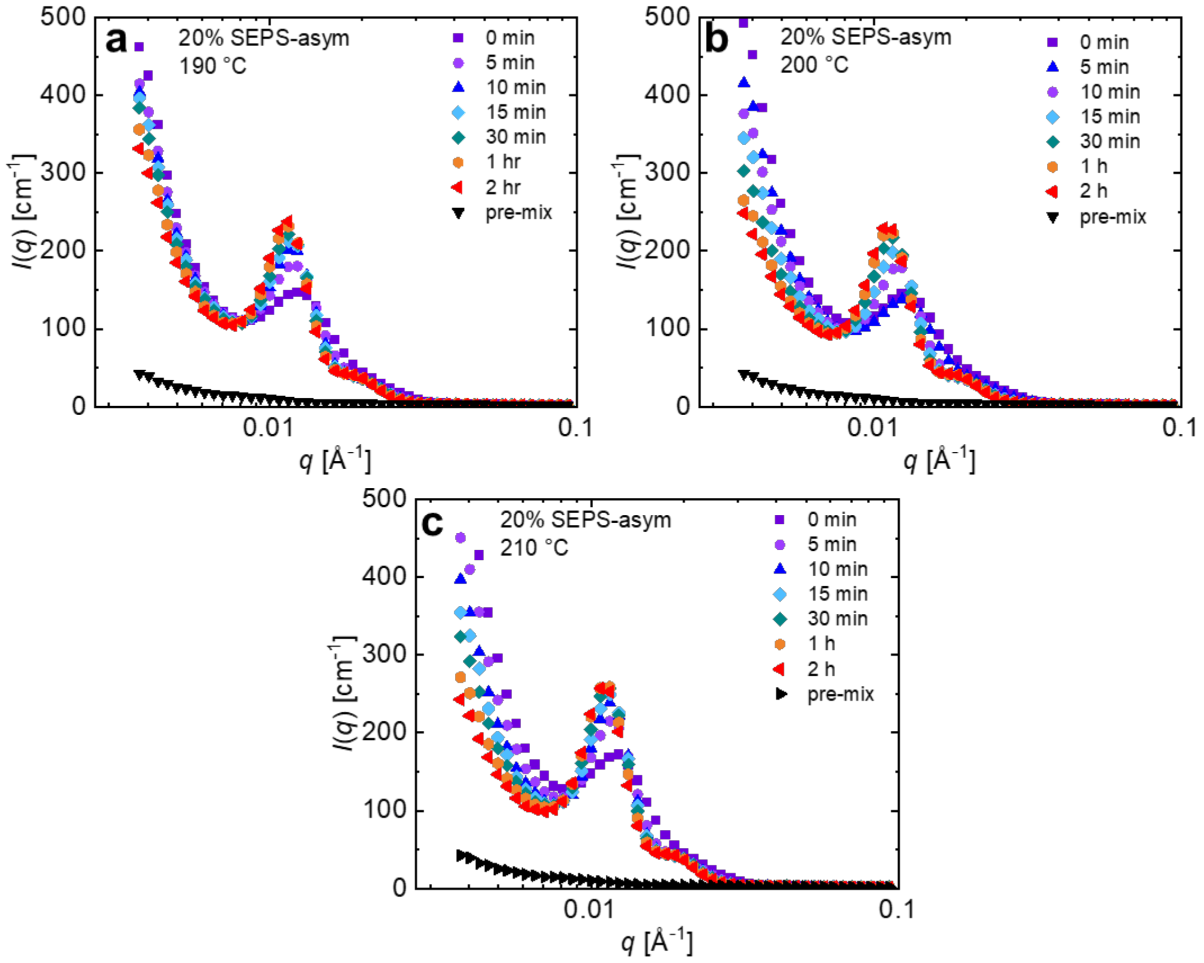

Figure S11. 1D TR-SANS traces for 20% SEPS-asym run at three different temperatures. All measurements were run with a 15 m sample-to-detector distance.

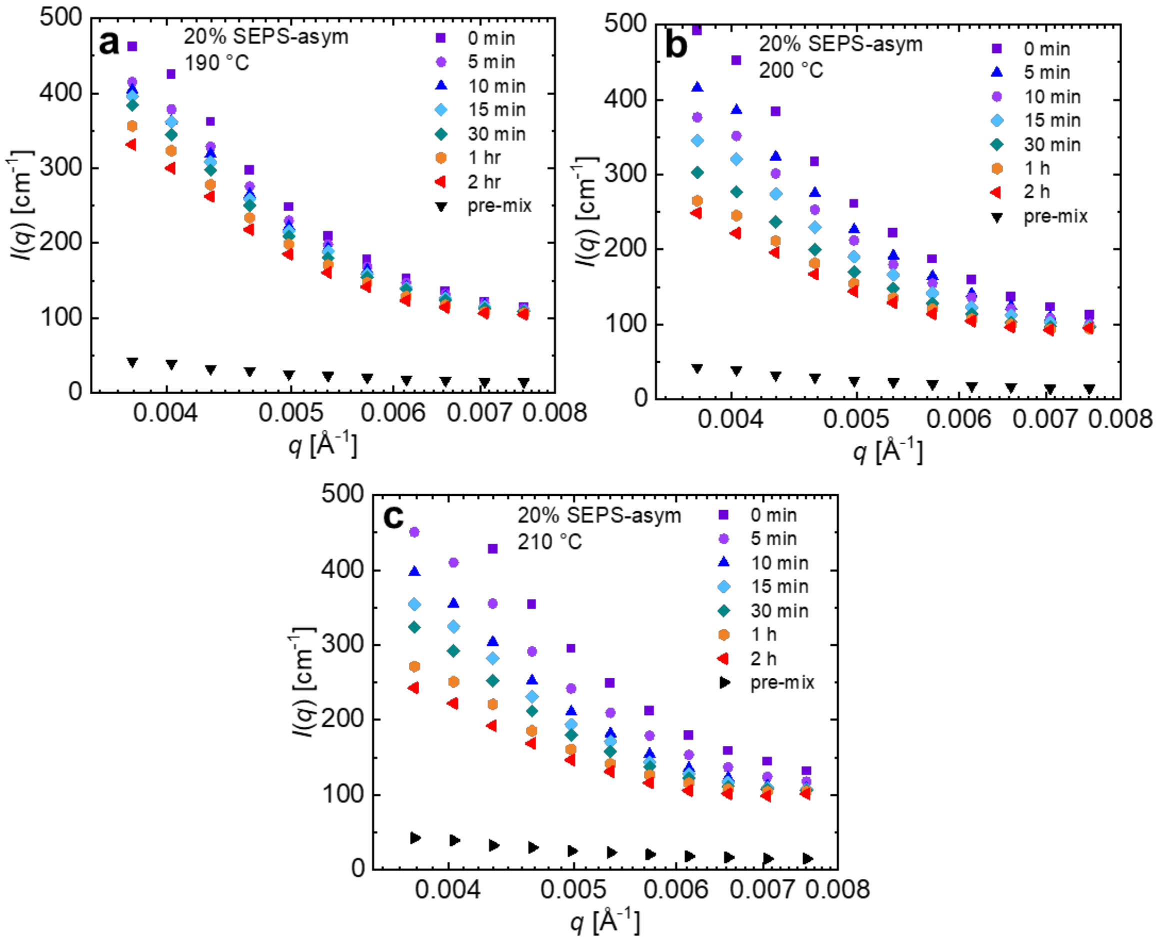

Figure S12. 1D TR-SANS traces for 20% SEPS-asym shown in Figure S11, zoomed in to focus on the q-region used for $R(t)$ analysis.

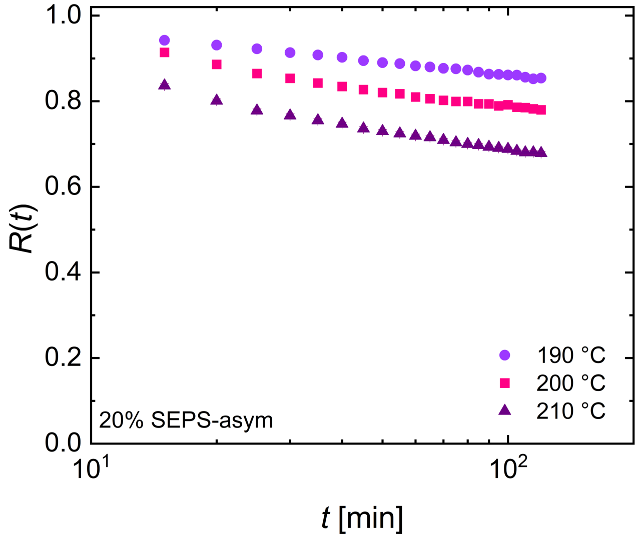

Figure S13. $R(t)$ curves for 20% SEPS-asym at three different temperatures. Error bars are included but are smaller than the symbols.

S5. $R(t)$ sensitivity and error analysis

Error bars are included on all the $R(t)$ curves, although some bars are not visible as they are smaller than the symbols. These errors were calculated by propagating the error associated with various intensity integrals using the following equation:

$$\delta R(t) = \left(\left[\frac{\partial R(t)}{\partial I(t)} \cdot \delta I(t)\right]^2 + \left[\frac{\partial R(t)}{\partial I(\infty)} \cdot \delta I(\infty)\right]^2 + \left[\frac{\partial R(t)}{\partial I(0)} \cdot \delta I(0)\right]^2\right)^{1/2} \quad \text{(S2)}$$

where $\delta I(t)$, $\delta I(\infty)$, and $\delta I(0)$ are the errors associated with $I(t)$, the pre-mixed sample $I(\infty)$, and the post-mixed sample $I(0)$, respectively.[5]

$R(t)$ curves presented in the main text were determined by integrating $I(q)$ in the low q regime. This region extended from where the impact of the beamstop was not significant (0.004 Å^{-1} for the 15 m setting and 0.008 Å^{-1} for the 7 m setting) up to the local minimum in the intensity prior to the primary peak in the structure factor (0.01 Å^{-1} for SEPS-sym and 0.008 Å^{-1} for SEPS-asym). Restricting to this region has several potential benefits. For particles with non-uniform scattering length densities, such as the post-mixed samples measured with SANS, the scattering intensity is defined by Eqn S3.

$$I(q) = (\Delta\rho)^2 v^2 z^2 N P(q) \left\{1 + \frac{|F(q)|^2}{P(q)}(S(q) - 1)\right\} \quad \text{(S3)}$$

where v is the volume of a scattering center, z is the number of scattering centers per particle, N is the number of particles, and $F(q)$ is the form factor amplitude. Note that for solid particles with uniform scattering length density, $|F(q)|^2 = P(q)$ and Eqn S1 holds.

As shown in Figure S5, $P(q)$ is nearly constant within this low q regime and the magnitude of $S(q)$ is very small. As a result, from eqn. S3,a $I(q)$ is fairly insensitive towards changes in either $P(q)$ or $S(q)$ as a function of time and therefore changes in $I(q)$ are almost entirely due to changes in scattering contrast, $(\Delta\rho)^2$, as is desirable for extracting $R(t)$. Alternatively, at higher q (as in the range for form factor fitting with SAXS), the structure factor approaches one and is nearly constant, but the form factor has many features defined by the size and distribution of chains in individual micelles. Changes in the configuration of chains *i.e.*, loop to bridge rearrangements, could impact the form factor and it could be difficult to deconvolute these effects from changes in contrast due to chain exchange. Using the full q range includes contributions from the primary structure factor peak. As shown in the raw SANS data, this peak sharpens and increases in intensity during the TR-SANS measurement, which could again be convoluted with $R(t)$. $R(t)$ curves for each of these q regions with the SEPS-sym sample are shown in Figure S14. As shown, the results are not extremely sensitive to the q region in these samples, but do lead to minor quantitative changes.

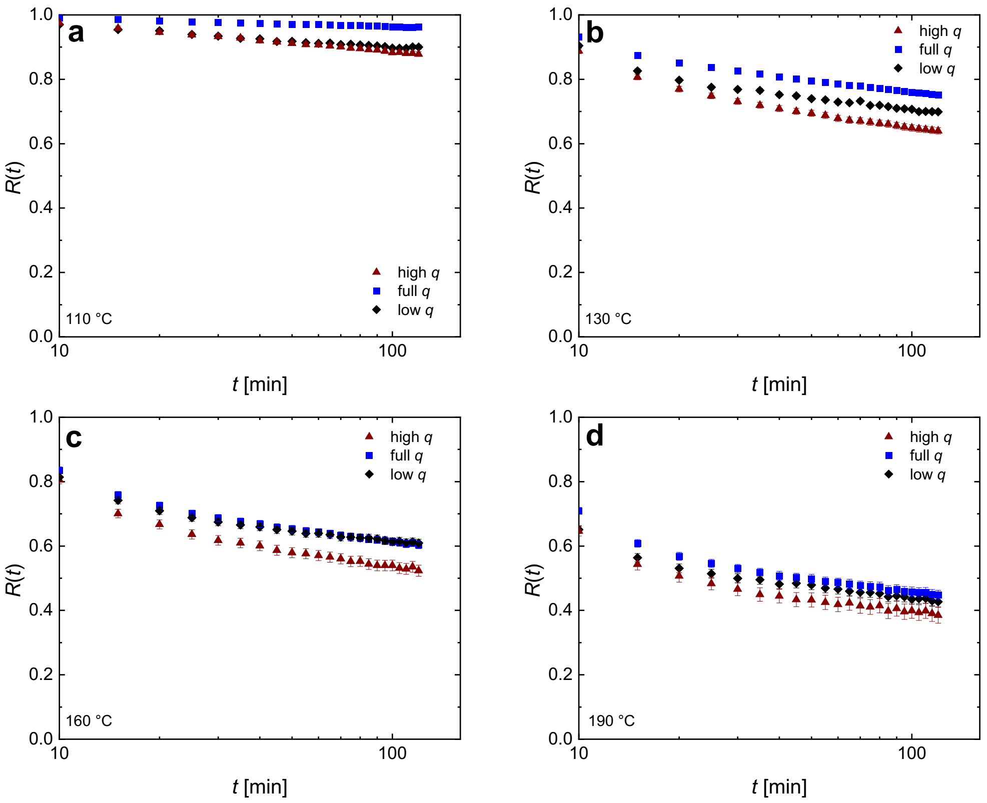

Figure S14. $R(t)$ curves calculated for SEPS-sym solutions using three different q regions for integration. The "low q" was used in the main text, the "full q" included the entire accessible q range, and the "high q" included the data above the inflection point after the primary structure factor peak.

S6. Preparation and implications of flower-like micelles in post-mixed samples

In very dilute solutions, loop formation is favored as a non-uniform distribution of micelles and significant corona-block stretching is necessary for bridges to form. To prepare samples for TR-SANS measurements, a cosolvent method was used. Dilute solutions of each each triblock polymer were prepared using 95% pentane and 5% dichloromethane as a cosolvent with squalane. The cosolvent was then removed at reduced temperature with dynamic vacuum (cold evaporation). To assess the occurrence of chain exchange and bridge formation during these evaporation steps, dynamic light scattering (DLS) was used.

First, solutions of 0.25 wt% SPS-1 in the cosolvent mixture were measured using DLS. This concentration is lower than that used during sample preparation, but is beneficial for this study, as nearly all bridge formation is eliminated, allowing for the measurement of isolated flower-like micelles. These pre-evaporation solutions exhibited a single relaxation mode, which was fit to a second cumulant expansion (Figure S15a and c). The cosolvents were then removed using the cold evaporation procedure to form a 20 wt% solution in squalane, before being re-diluted with squalane to 0.25 wt%. The resulting micelles were similarly sized and exhibited a

single relaxation mode (Figure S15b and d). Note that the large difference in relaxation rates for the two solutions is due to the difference in solvent viscosity. In contrast, 20 wt% solutions that were annealed at 160 °C for 4 h could not be diluted with squalane at room temperature, and instead swelled to a modest degree, with much of the excess squalane remaining separated, demonstrating substantial bridge formation after annealing (Figure S16).”

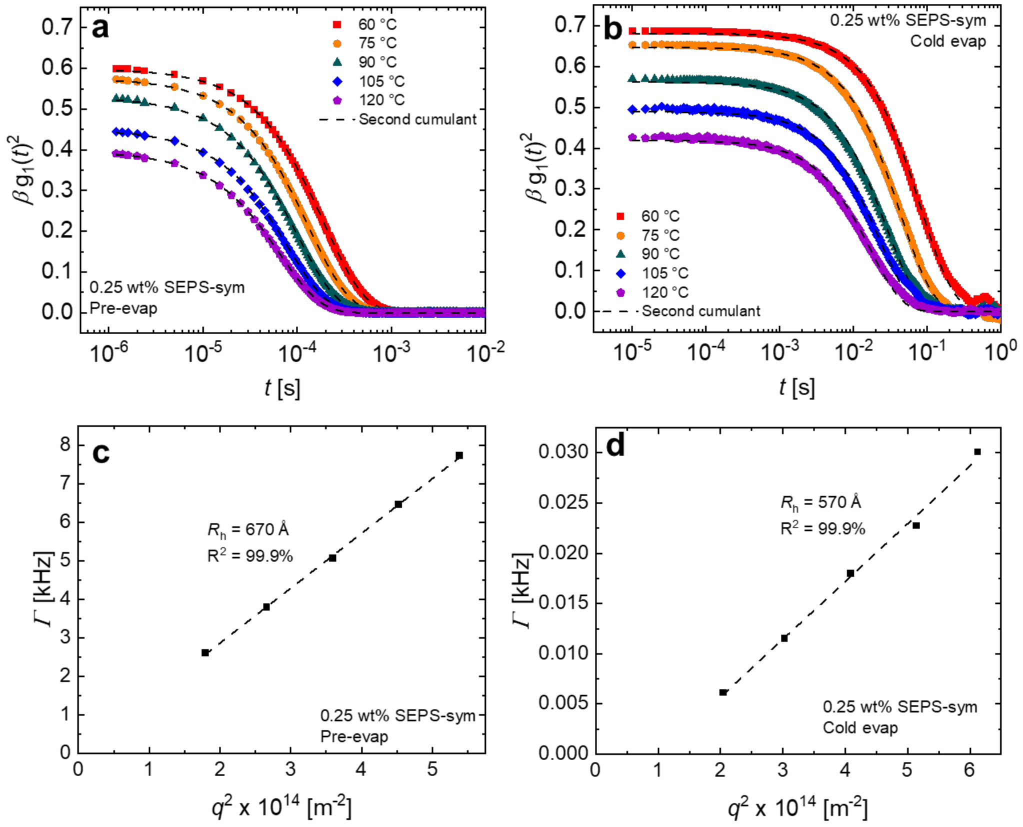

Figure S15. Intensity autocorrelation functions for 0.25 wt% SPS-1 (a) pre-evaporation and (b) after cold-evaporation and re-dilution with squalane. Corresponding plots showing the diffusive nature of the process and estimated R_h values for (c) pre-evaporation and (d) cold-evaporation samples.

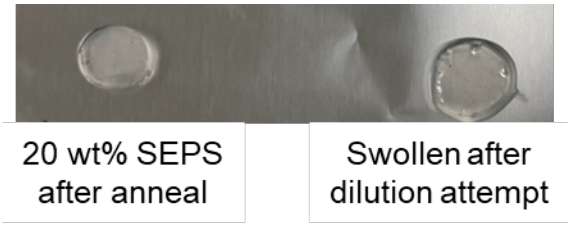

Figure S16. Pictures of 20 wt% SEPS film after being annealed and then after attempting to redissolve/dilute in squalane. As shown, the film swelled a little, but did not dissolve.

While the equilibrium bridge fraction in this system is not known, previous reports estimate triblocks within a percolated network typically form ~40% bridges and 60% loops (though the exact values vary). Initially, samples for TR-SANS measurements have only loops and over the course of the measurement, a closer-to-equilibrium fraction of bridges evolves. Until the longer core block escapes, bridge formation can only occur through movement of the shorter block to neighboring micelles. Bridge formation between two micelles of the same core identity (dPS or hPS) would not change the overall scattering contrast. Assuming that the dPS and hPS micelles are initially well mixed, for any given micelle approximately 50% of its neighbors contain the other isotope. Consequently, if 40% of chains formed bridges, when one end of 50% of those chain moved, and 50% of neighbors are of a different identity, achieving an equilibrium bridge fraction would result in only a ~10% change in $R(t)$. This concept is illustrated in Figure S17.

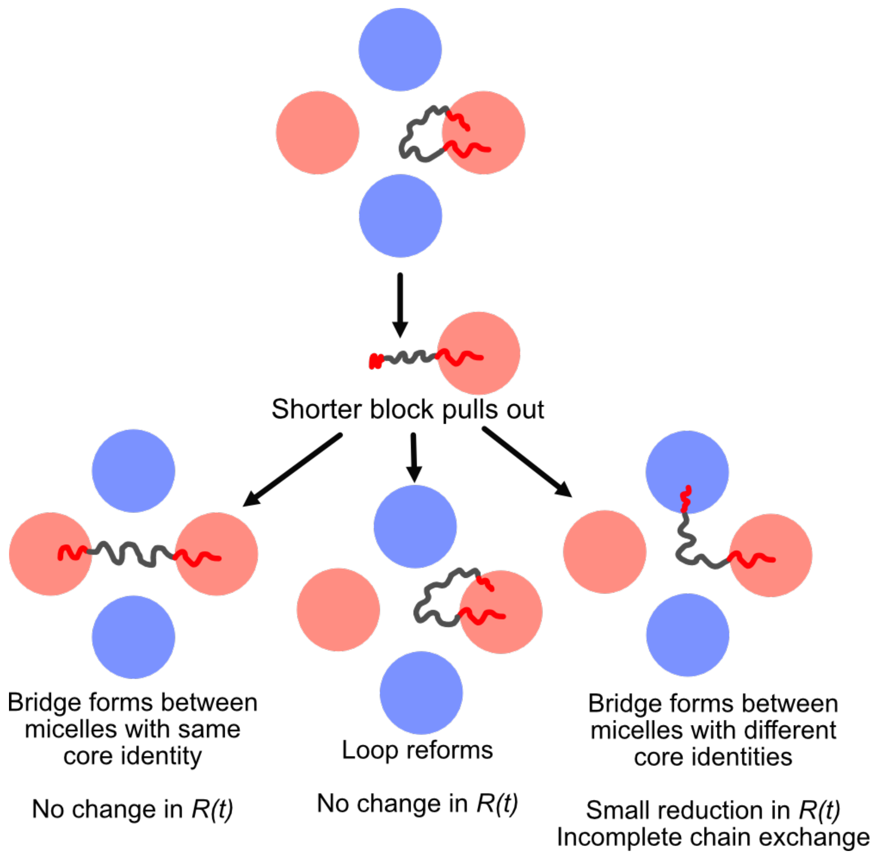

Figure S17. Schematic illustrating the possible movements of the shorter block prior to long block pullout. As shown, only bridge formation between micelles of dissimilar identities results in a reduction in $R(t)$.

S7. Estimation of chain diffusion time

Frequency sweeps of 15 wt% PEP (100 kDa synthesized in-house with anionic polymerization of polyisoprene and sequential hydrogenation) in squalane were performed on an

ARES-G2 rheometer using a strain amplitude of 1%. This concentration is nearly identical to the effective concentration of PEP within the matrix in the 20 wt% SEPS samples. Data were collected at temperatures between –60 and 100 °C and shifted using tTs to generate a master curve (Figure S18a). The shift factors were well-described by the WLF relationship (Figure S18b). From this master curve, the reptation time (τ_{rep}) was estimated. Using the reptation model, the diffusion coefficient within the matrix was estimated according to eqn. S4.

$$Nb^2 = 6D_{\mathrm{t}}\tau_{\mathrm{rep}} \tag{S4}$$

where N is the number of repeat units in the PEP chain and b is the statistical segment length of PEP (8 Å).[6,7]

The timescale for diffusion across the distance between two micelles was then estimated using eqn. S5, where d_{nn} is the distance between micelles, approximated as $2*R_{\mathrm{HS}}$ from the SAXS fits.

$$\tau_{diff} = \frac{(d_{nn})^2}{D_t} \tag{S5}$$

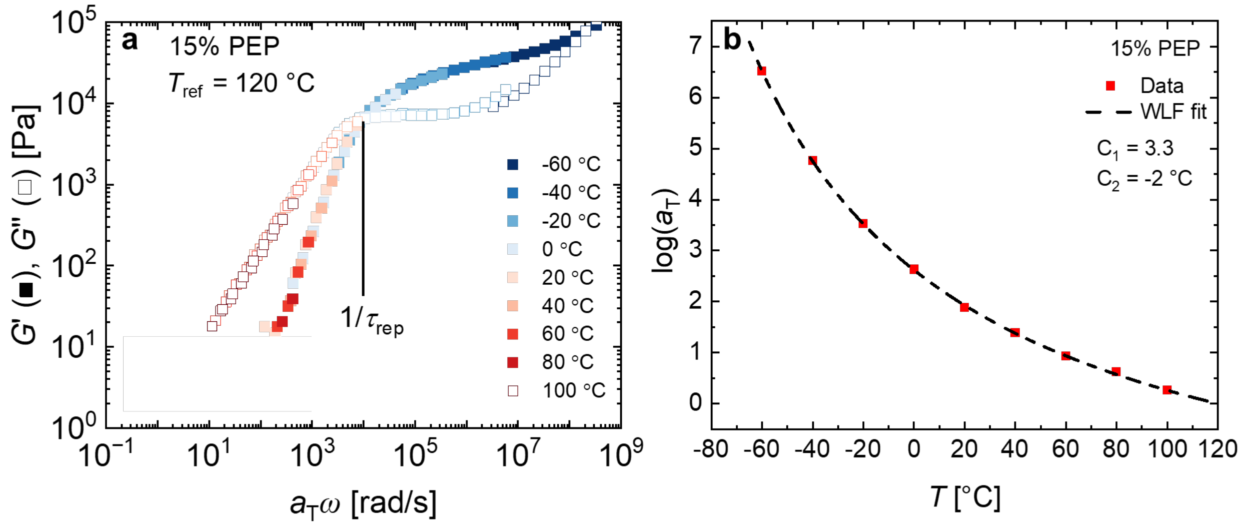

Figure S18. (a) Frequency sweep master curve of 15 wt% 100 kDa PEP homopolymer in squalane. Measurements were shifted using tTs, and the reptation time (τ_{rep}) was extracted. (b) Shift factors used to generate the master curve. As shown, they follow the WLF form.

S8. Sensitivity of core-block dispersity and corona-block stretching

The model describing the *R(t)* and *G(t)* data for 20% SPS-sym presented in the main text (Figure 4) uses a nominal core-block dispersity ($Đ_{\mathrm{core}}$) of 1.06 and assumes that both end-blocks

fall into a single Schulz-Zimm distribution. As shown in Figure S19 and discussed previously, the "exponential of an exponential" dependence of the $R(t)$ model on N_{core} makes the results hypersensitive to $Đ_{core}$.[8] Further, the refined triblock model relies even more heavily on $Đ_{core}$ to generate revised distribution functions. Decreasing $Đ_{core}$ reduces the difference in timescale between the stress relaxation and TR-SANS models and sharpens the decay function. With sequential anionic polymerization, there is likely a slightly higher degree of asymmetry between the two blocks which would further exacerbated the differences between $P(N_{core,S})$ and $P(N_{core,L})$ which are not accounted for in these distributions. Degradation and aliquot SEC profiles of the PS end-blocks (Figure S2) suggest that this effect should be minimal, but even small deviations can dramatically alter the modelled $R(t)$ profile.

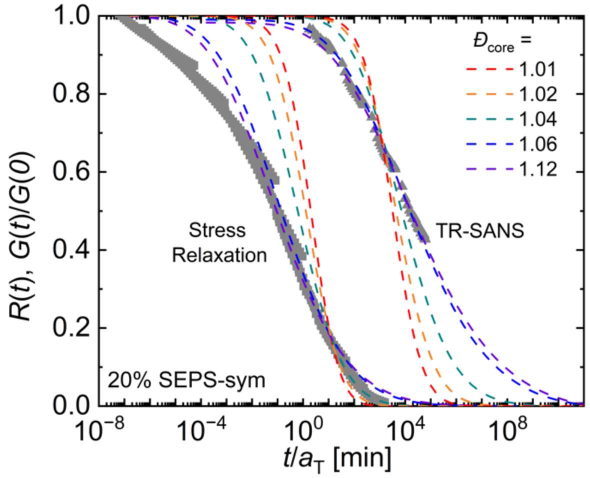

Figure S19. Normalized stress relaxation and TR-SANS $R(t)$ curves for 20% SEPS-sym. Overlayed are models describing these data with varying degrees of $Đ_{core}$ for the full core-block distribution.

The stress relaxation model also incorporates a second entropic term attributed to the relief in corona chain stretching upon bridge breakage. In the main text, the value of s_{corona} was approximated as 2.0 based on the best fit to the stress relaxation data. As shown in Figure S20, the model profile varies substantially for different values of s_{corona}. Little weight should be placed on these exact values, however, as the fitted value for s_{corona} would change with minor changes to $P(N_{core,S})$ due to asymmetries from sequential anionic polymerization, as discussed above.

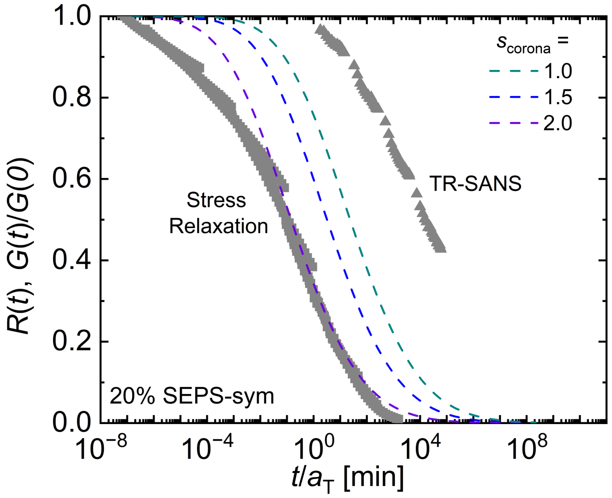

Figure S20. Normalized stress relaxation and TR-SANS $R(t)$ curves for 20% SEPS-sym. Overlayed are models describing the stress relaxation data with varying values of s_{corona}

Fitting of SAXS data can also be used to approximate a reasonable value for s_{corona}. The spherical form factor was used to obtain a value for the radius of the PS core, R_{core}. From R_{core}, the mean aggregation number, N_{agg}, was approximated by assuming that the core was composed of pure PS. Based on the volume fraction of polymer added and the molar mass determined using SEC, the number of chains per unit volume, n_{chains}, was determined. Employing geometric arguments, the average distance between micelles, d, was approximated as

$$d = 2\left(\frac{3N_{agg}}{4\pi n_{chains}}\right)^{1/3} \quad \text{(S6)}$$

and the stretching distance of the corona was approximated as

$$h = d - 2R_{core} \quad \text{(S7)}$$

These values resulted in a value for s_{corona} of 1.5.

S9. Stress relaxation of 20% SEPS-asym

A master curve of the stress relaxation data for 20% SEPS-asym is shown in Figure S21. Using the same method described in the main text, the curve was fit to a stretched exponential function. From this fit, <τ> was estimated as 235 min at 120 °C, which is the same order-of-magnitude as the SEPS-sym estimate of 105 min.

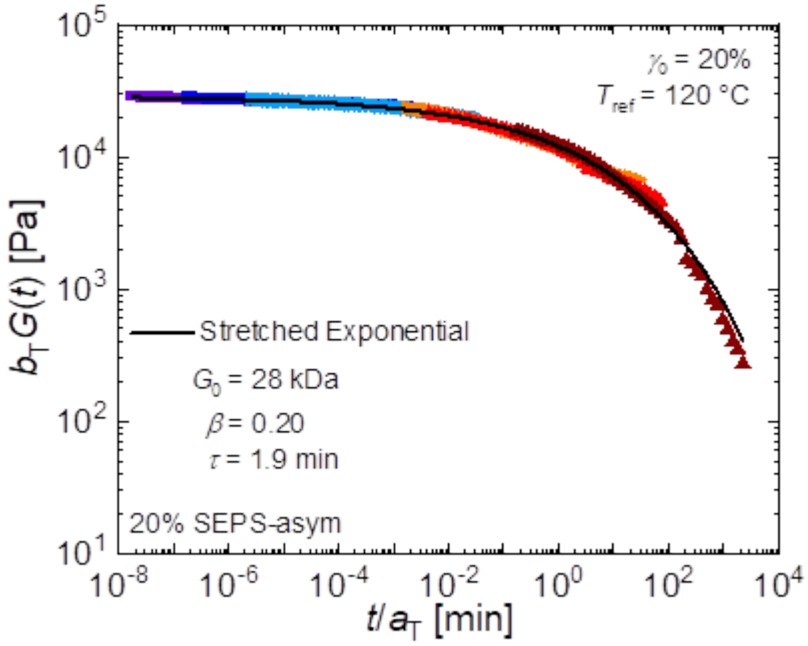

Figure S21. Master curve of stress relaxation data for 20% SEPS-asym shifted to a reference temperature of 120 °C. Fits to a stretched exponential were used to extract an average relaxation time.

References

(1) Mahanthappa, M. K.; Bates, F. S.; Hillmyer, M. A. Synthesis of ABA Triblock Copolymers by a Tandem ROMP−RAFT Strategy. *Macromolecules* **2005**, *38* (19), 7890–7894.

(2) Doucet, M.; Cho, J. H.; Alina, G.; Attala, Z.; Bakker, J.; Bouwman, W.; Butler, P.; Campbell, K.; Cooper-Benun, T.; Durniak, C.; Forster, L.; Gonzalez, M.; Heenan, R.; Jackson, A.; King, S.; Kienzle, P.; Krzywon, J.; Murphy, R.; Nielsen, T.; O'Driscoll, L.; Potrzebowski, W.; Prescott, S.; Ferraz Leal, R.; Rozyczko, P.; Snow, T.; Washington, A. SasView Version 5.0.4, 2021.

(3) Pedersen, J.S. Structure Factors Effects in Small-Angle Scattering from Block Copolymer Micelles and Star Polymers. *The Journal of Chemical Physics* **2001**, *114* (6), 2839-2846.

(4) Hamley, I.W.; Castelletto, V. Small-Angle Scattering of Block Copolymers in the Melt, Solution, and Crystal States. *Progress in Polymer Science* **2004**, *29* (9), 909-948.

(5) Wang, E.; Lu, J.; Bates, F. S.; Lodge, T. P. Effect of Corona Block Length on the Structure and Chain Exchange Kinetics of Block Copolymer Micelles. *Macromolecules* **2018**, *51* (10), 3563–3571.

(6) Rubinstein, M.; Colby, R.H. *Polymer Physics*; Oxford University Press: Oxford, 2003.

(7) Lodge, T. P.; Hiemenz, P. C. *Polymer Chemistry*; CRC Press: Boca Raton, FL, 2021.

(8) Choi, S.-H.; Lodge, T. P.; Bates, F. S. Mechanism of Molecular Exchange in Diblock Copolymer Micelles: Hypersensitivity to Core Chain Length. *Phys. Rev. Lett.* **2010**, *104* (4), 047802.
